# Sampling across large-scale geological gradients to study geosphere–biosphere interactions

**DOI:** 10.3389/fmicb.2022.998133

**Published:** 2022-10-31

**Authors:** Donato Giovannelli, Peter H. Barry, J. Maarten de Moor, Gerdhard L. Jessen, Matthew O. Schrenk, Karen G. Lloyd

**Affiliations:** ^1^Department of Biology, University of Naples “Federico II”, Naples, Italy; ^2^Institute of Marine Biological Resources and Biotechnologies, National Research Council, CNR-IRBIM, Ancona, Italy; ^3^Department of Marine and Coastal Science, Rutgers University, New Brunswick, NJ, United States; ^4^Marine Chemistry and Geochemistry Department, Woods Hole Oceanographic Institution, MA, United States; ^5^Earth-Life Science Institute, Tokyo Institute of Technology, Tokyo, Japan; ^6^Observatorio Volcanológico y Sismológico de Costa Rica (OVSICORI), Universidad Nacional, Heredia, Costa Rica; ^7^Department of Earth and Planetary Sciences, University of New Mexico, Albuquerque, NM, United States; ^8^Instituto de Ciencias Marinas y Limnológicas, Universidad Austral de Chile, Valdivia, Chile; ^9^Center for Oceanographic Research COPAS COASTAL, Universidad de Concepción, Concepción, Chile; ^10^Department of Earth and Environmental Sciences, Department of Microbiology and Molecular Genetics, Michigan State University, East Lansing, MI, United States; ^11^Microbiology Department, University of Tennessee, Knoxville, TN, United States

**Keywords:** subsurface biosphere, geosphere–biosphere coevolution, geomicrobiology, large-scale, hot springs

## Abstract

Despite being one of the largest microbial ecosystems on Earth, many basic open questions remain about how life exists and thrives in the deep subsurface biosphere. Much of this ambiguity is due to the fact that it is exceedingly difficult and often prohibitively expensive to directly sample the deep subsurface, requiring elaborate drilling programs or access to deep mines. We propose a sampling approach which involves collection of a large suite of geological, geochemical, and biological data from numerous deeply-sourced seeps—including lower temperature sites—over large spatial scales. This enables research into interactions between the geosphere and the biosphere, expanding the classical local approach to regional or even planetary scales. Understanding the interplay between geology, geochemistry and biology on such scales is essential for building subsurface ecosystem models and extrapolating the ecological and biogeochemical roles of subsurface microbes beyond single site interpretations. This approach has been used successfully across the Central and South American Convergent Margins, and can be applied more broadly to other types of geological regions (i.e., rifting, intraplate volcanic, and hydrothermal settings). Working across geological spatial scales inherently encompasses broad temporal scales (e.g., millions of years of volatile cycling across a convergent margin), providing access to a framework for interpreting evolution and ecosystem functions through deep time and space. We propose that tectonic interactions are fundamental to maintaining planetary habitability through feedbacks that stabilize the ecosphere, and deep biosphere studies are fundamental to understanding geo-bio feedbacks on these processes on a global scale.

## Introduction

Earth’s continental and oceanic crust contain one of the largest microbial ecosystems on the planet (Kallmeyer et al., 2012; Magnabosco et al., 2018). This subsurface microbial biosphere is essential to global biogeochemical cycles because it alters the redox state of the crust and affects the distribution of minerals, gases, and organic matter through deep time (D’Hondt et al., 2019). While subsurface microbiology is often studied over small spatial scales (sampling sites meters to a few kilometers apart), or over short time scales during laboratory experiments, crustal ecosystems likely operate on tectonic scales of hundreds of kilometers and millions of years. Despite their importance to planetary processes and volatile cycling through deep time, much less is known about subsurface microbial ecosystems than surface microbes. This is largely due to the difficulty in accessing deep subsurface environments.

Direct sampling of the subsurface is fundamental, not only for obtaining samples that can be used to grow and study organisms in a laboratory, but also for conducting studies on diverse populations as they occur naturally. Such in situ studies are important because many microbes in the subsurface belong to microbial groups that are resistant to many or all culturing efforts—meaning that they are not amenable to pure culture experimentation (e.g., Rappé and Giovannoni, 2003; Lloyd et al., 2018; Steen et al., 2019). Even if laboratory cultures are available, the physiology and ecological roles of microbes may differ in the natural, taxonomically-diverse, often energy-limited environment relative to laboratory conditions, as originally proposed by Winogradsky (1923). Direct access to the subsurface is normally obtained through scientific drilling. Major drilling operations occur through the International Ocean Discovery Program (IODP-US), which was formerly the Integrated Ocean Drilling Program (IODP), the Ocean Drilling Program (ODP), and the Deep Sea Drilling Program (DSDP), as well as the European Consortium for Ocean Research Drilling (ECORD), Japan’s Integrated Ocean Drilling program (IODP-J), and the International Continental Scientific Drilling Program (ICDP). These programs have provided unprecedented access to subsurface ecosystems. However, deep drilling projects are expensive and infrequent (Orcutt and Edwards, 2014), and require complex logistical operations and a long lead time (sometimes many years) before the actual sampling. Thus drilling makes it difficult to sample more than a handful of subsurface sites in a single study, or to collect new samples quickly to react to new scientific advances and opportunities created by geologically dynamic events (e.g., earthquakes or eruptions). The large costs, duration of fieldwork, and organizational requirements can also be a barrier to entry for early career researchers, or researchers working in countries with limited research and development funding. Access to commercial or scientific mines and mining sites are another fruitful approach to studying the deep subsurface biosphere (e.g., Chivian et al., 2008), but site selection is limited to existing infrastructure, i.e., samples are determined by where the mines happen to be. Besides drilling and access to existing subsurface facilities there is a need for a more nimble approach to obtaining subsurface samples.

Subseafloor hydrothermal vent fluids have been used to sample deep subsurface life as it is flushed out of the subsurface, rather than by drilling down to it (Deming and Baross, 1993). Here, we expand this approach to large scales and by assessing the degree of mixing with the surface, accelerate exploration of the subsurface biosphere and enable large-scale studies of biosphere-geosphere coupling of subsurface microbial ecosystems across regional or global geological features. Typically, microbiological studies of terrestrial hot springs or oceanic hydrothermal vents study either variations across a transect at a single site or compare a handful of sites to one another or to a background reference site (Giovannelli et al., 2013; Reveillaud et al., 2016; Brazelton et al., 2017). We propose instead to sample many natural seeps across a geological gradient to map out broad-scale ecological features in a large area of the subsurface. The purpose of this sampling approach is not to replace direct sampling strategies or careful transect studies across a single site, but to add to the arsenal of sampling approaches that can greatly expand our ability to sample microbial populations across large spatial regions. We have successfully employed this approach to study biosphere-geosphere feedbacks across the convergent margin in Northern and Central Costa Rica (Barry et al., 2019; Fullerton et al., 2021; Basili et al., 2022; Rogers et al., 2022) and Argentina (Cascone et al., 2021). This approach is flexible, interdisciplinary, can be widely applied to diverse settings, and is orders of magnitude cheaper and faster than drilling. Here we describe the advantages and drawbacks of this approach, as well as provide suggestions based on experience for how other researchers can employ a similar approach in their own work. When integrated with targeted drilling projects, our approach can provide unprecedented insights into the interactions between subsurface ecosystems and geological processes at large spatial scales.

### Deeply-sourced seeps as windows into the subsurface

Natural seep fluids have previously been proposed as windows into the subsurface biosphere both at deep-sea hydrothermal vents (e.g., Deming and Baross, 1993; Summit and Baross, 2001; Anderson et al., 2011) and in terrestrial settings (e.g., Meyer-Dombard et al., 2005; Hall et al., 2008; Hou et al., 2013; Colman et al., 2016; Crossey et al., 2016; Uribe-Lorío et al., 2019). Typically, high temperature fluids have been interpreted as ascending quickly from depth (i.e., minimal surface mixing) and thus representative of the subsurface. Fluids and volatiles released at lower temperature sites, such as those found in the forearc of convergent margins or in low temperature pools at the margin of large calderas are typically ignored, as the degree of mixing of the fluids with surface derived waters might be difficult to assess. By focusing on high temperature sites, the information relevant to the subsurface microbial communities is limited by the geothermal gradient, as higher temperature sites (> 122°C) are not amenable to life (Merino et al., 2019). By combining interdisciplinary co-located measurements it is possible instead to assess the source of volatiles and deconvolve the degree of mixing with the surface providing new insight into deeply-sourced seeps and thus the deep biosphere. Additionally, focusing on the gradients in features of the seeping fluids across the deeply-sourced seeps has the additional effect of changing the scale at which geochemical gradients can be investigated. In this context, the focus of the sampling moves from hot springs sensu stricto to features that channel surface deep fluids, regardless of their temperature, conductivity or pH.

**Figure 1:**
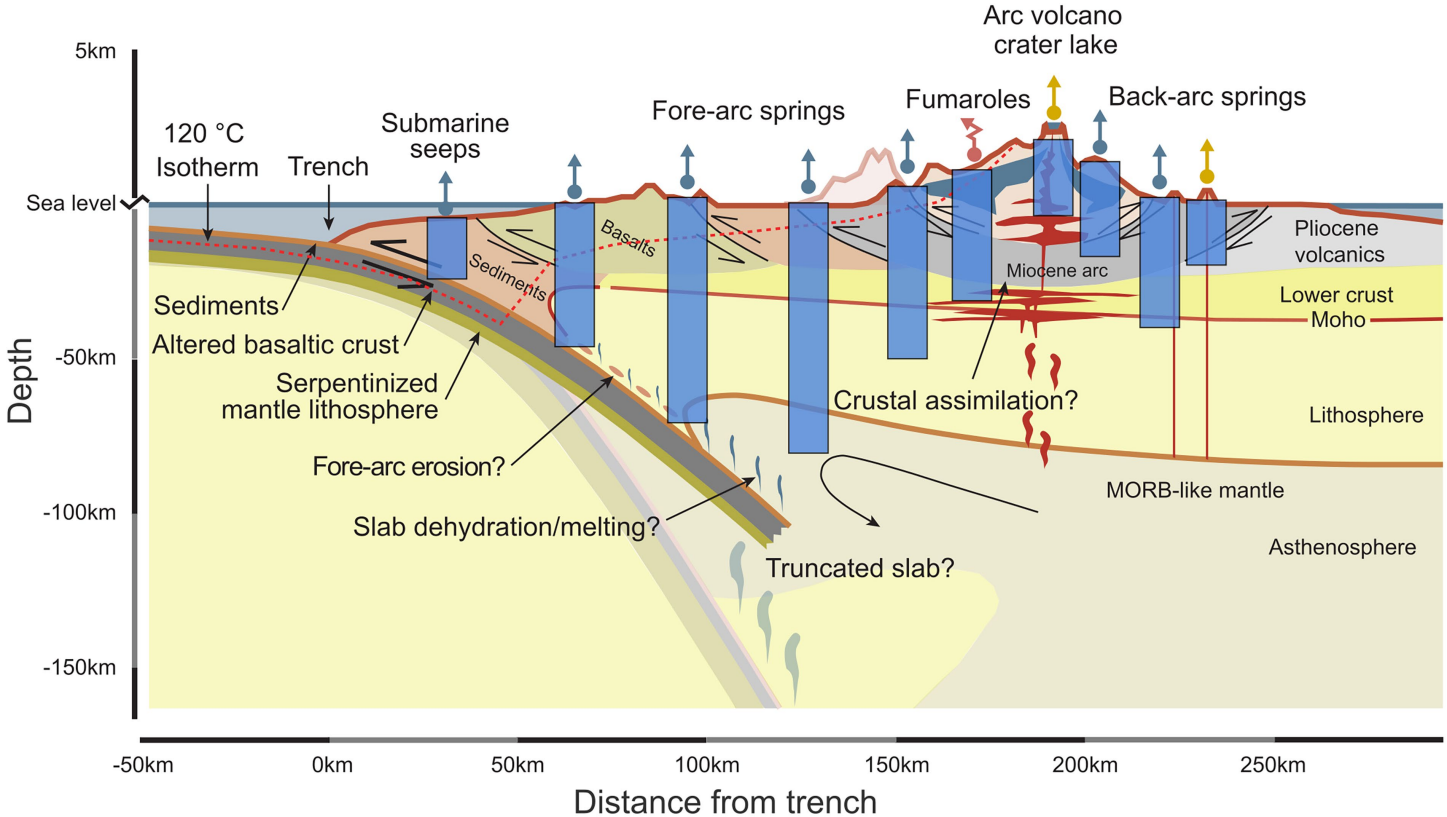
Sampling deeply sourced seeps as windows into the subsurface across geological provinces can help to identify biological interactions with large-scale geological processes. Each deeply-sourced seep (represented by a straight upward arrow for fluids, teal for springs, sand for volcanic crater waters, or by a jagged red arrow for gases) gives access to an integrated view of the source and processes occurring at depth (light blue rectangles). Transects across large-scale geological gradients can be obtained using this approach. Modified after Giovannelli et al. (2020).

We therefore define a broad category of features resulting from deep fluids ascending to the surface called ***deeply-sourced seeps*** (Box 1). Deeply-sourced seeps are the surface expression of deep fluids, either as water or gases, that transport volatiles from depth (Figure 1). They are not necessarily associated with high temperature fluids, and thus include lower temperature manifestations and diffuse degassing areas (Crossey et al., 2016). In order to identify and use deeply-sourced seeps as windows into the subsurface, it is essential to adopt an experimental approach that distinguishes between samples that represent the biology and geochemistry of the subsurface and those that show strong mixing with surface derived volatiles and microbial communities. Our approach combines assessments of proxies of subsurface vs. surface material (e.g., He and C isotopes; Crossey et al., 2016; Barry et al., 2019), as well as comparing communities across large spatial gradients to assess which parts of the microbial community correlate with deep subsurface features vs. surface features (Crossey et al., 2016; Cascone et al., 2021; Fullerton et al., 2021; Basili et al., 2022; Rogers et al., 2022). These proxies include dissolved and gaseous inorganic compounds, isotopic composition (e.g., Δ^14^C and δ^13^C of dissolved organic and inorganic carbon; Lang et al., 2018; Fullerton et al., 2021) or biomolecules that can indicate the degree of exposure the fluids had with the surface or surface derived shallow fluids (e.g., photosynthetic pigments and genetic signatures of phototrophy or obligate aerobic metabolisms; Table 1).

**Table 1:** Parameters that can be used to track the relative contribution of surface and subsurface processes to the samples deeply-sourced seeps.

Variable measured	Sampled phase	Sources tracked	Typical deep signature
^3^He/^4^He	Free and dissolved gases	Crustal vs. mantle contribution to volatiles	^3^He/^4^He value above 0.5 R_A_ (relative to air) contain possible mantle contributions, with value around 5–6 R_A_for arc volcanoes, 8 R_A_for pure upper mantle and > 9 R_A_ for deeper mantle contributions
^4^He/^20^Ne and ^40^Ar/^36^Ar	Free and dissolved gases	Mixing with air or air saturated fluids	Values > 10 for ^4^He/^20^Ne and > 350 for ^40^Ar/^36^Ar signal minimal interaction with air or air saturated fluids
N_2_–He–Ar systematics	Free and dissolved gases	Crustal vs. mantle contribution to volatiles and interaction with atmospheric sources	High He/Ar indicates deep source (mantle or crust), high N_2_/He reflects input of subduction fluids, atmospheric contribution identified by N_2_/Ar of 40–80.
Aqueous major ion species	Fluids	Gas–water–rock interactions and mixing of different reservoirs	Ternary plots of major ions can be used to identify gas–water–rock interactions and distinguish deep waters from meteoric derived waters. Compositions depend on the type of tectonic setting or geological process investigated
Dissolved O_2_ and O_2_/Ar	Fluids	Recent interaction with air or air saturated waters	Anoxic waters are indicative of low to absent mixing with surface derived fluids and air intrusion
^14^C in fluids	Fluids	Interaction with surface derived fluids	Radiocarbon dead waters indicate an age of > 50,000 years and no interaction with recent surface waters
δD_2_, δ^18^O of H_2_O	Fluids	Evaporation, water–rock interactions, mixing with magmatic waters or rock-equilibrated waters	Deviation form the local meteoric water line can be used to identify processes acting on the sample fluids and their potential interaction with deep fluids
δ^13^C of different carbon reservoirs	Fluids, sediments and surrounding soils	Deep origin of the different carbon reservoirs	Atmospheric CO_2_ has a signature around-8 ‰, while pure mantle derived CO_2_ has a signature of-5 ‰. Photosynthetic derived organic carbon has a value around-25 ‰. Differences between the dissolved inorganic carbon and dissolved organic carbon of fluids *vs* the total organic carbon in sediment and surrounding soils can be used to infer a deep source of the inorganic and/or organic carbon in the fluids
Photosynthetic pigments (e.g., Chlorophyll-a)	Fluids	Recent exposure to surface environments	Chlorophyll-a is a labile compound with a short half life. Its absence in the particulate fraction of fluids suggest no recent interaction or mixing with surface waters
Chloroplastic, Cyanobacterial or anoxygenic phototrophs sequences in 16S rRNA survey	Fluids and sediments	Absence of related sequences in fluids suggests no recent exposure to light	The absence of 16S rRNA sequences related to chloroplasts or known phototrophs suggest minimal mixing with surface derived waters and plant-derived detrital material
Oxygenic or anoxygenic photosynthesis genes in metagenomes	Fluids and sediments	Absence of related sequences in fluids suggests no recent exposure to light	The absence of sequences related to photosynthetic genes suggest minimal mixing with surface derived waters and plant-derived detrital material
Terminal oxidases utilizing oxygen as substrate in metagenomes	Fluids and sediments	Absence or limited presence of oxygen utilizing enzymes suggest the absence of recent mixing with surface derived oxygenated waters	The absence of sequences related to oxygen utilizing enzymes suggest minimal air contamination and mixing with surface derived waters

For example, mantle and crustal contributions to the volatile inventory can be tracked using a combination of helium isotope systematics (Hilton et al., 1993; Bekaert et al., 2021), while mixing with air saturated waters (i.e., surface waters) and water-rock interactions at depth can be tracked using a combination of N_2_–He–Ar systematics and aqueous geochemistry (e.g., Giggenbach, 1992). The exact nature of the proxy to be used depends on the tectonic setting under investigation and the type of samples collected (Table 1), and a multi-phase approach should be designed to accurately determine the degree of surface (i.e., atmospheric or shallow aquifers) contamination of the collected samples. Thus, suitable geochemical and biological tracers can be combined to the community structure and function of microbial populations across all the sites where there are sequencing data, to see which subpopulations correlate with subsurface geochemical and geological features (Crossey et al., 2016; Fullerton et al., 2021; Rogers et al., 2022).

In addition to using specific proxies to track subsurface–surface mixing, the type of sample collected (i.e., thermal feature or matrix), the sampling approach, and the mode of collection employed can significantly affect the quality of the data and environment (surface vs subsurface) that is ultimately represented. Typical work in hot springs, shallow-water hydrothermal vents, and sepentinizing settings looks at the interplay between subsurface and surface processes, often sampling the interface where deep fluids mix with surface oxidants (mainly atmospherically derived; Inskeep et al., 2005; Meyer-Dombard et al., 2005; Giovannelli et al., 2013; Sánchez-Murillo et al., 2014; Colman et al., 2016; Patwardhan et al., 2018;Colman et al., 2019b). In our approach, we attempt to minimize surface inputs by sampling deeply-sourced fluids as directly as possible. This is accomplished using the following multi-pronged field approach: (i) the seep is first identified using a combination of visual inspection and in situ temperature, conductivity and pH measurements, i.e., in the presence of several thermal features, the seeping fluids with the highest temperature, the highest or lowest pH, or highest salinity and flux are prioritized; (ii) with a minimum perturbation of the thermal feature and surroundings; a sterile non-reactive (glass or titanium) pipe/tube is inserted (> 30 cm deep) in the seep to catch the fluids before they mix with surface water or air; and (iii) the rate at which fluids are collected needs to be similar to the rate of natural seepage to avoid air/surface entrainment.

The quality of the sample collected can be assessed using a combination of geochemical tracers directly in the field or back in laboratory (e.g., dissolved O_2_, O_2_/Ar systematic, ^4^He/^20^Ne, see Table 1), while the degree of surface mixing can be assessed using a combination of diverse proxies. This approach has shown to minimize the possibility of mixing subsurface and surface fluids during sampling, both for geochemical (e.g., Barry et al., 2019) and microbiological studies (Cascone et al., 2021; Fullerton et al., 2021; Rogers et al., 2022). Additionally, sampling seep derived sediments and surrounding soils is also commonly done, to track surface interactions of the fluids with topsoils (Cascone et al., 2021; Fullerton et al., 2021).

### Collect samples across large spatial scales traversing geological features

Studying microbial communities across large spatial scales (spanning many kilometers or even the entire globe) has provided great insights into terrestrial and shallow hot springs microbial communities (Hou et al., 2013; Inskeep et al., 2013; Crossey et al., 2016; Power et al., 2018) as well as global or large-scale regional soil and oceanic water microbiome (Williamson et al., 2012; Sunagawa et al., 2015; Delgado-Baquerizo et al., 2018; Acinas et al., 2021). These studies have foregone the small-scale, local gradient approach in favor of large-scale patterns across gradients spanning entire ecosystems or regions of the globe. In contrast, a large number of subsurface studies have either focused on a handful of samples collected in close proximity (i.e., springs and hot springs close to each other; e.g., Emerson et al., 2016; Colman et al., 2019b; Trembath-Reichert et al., 2019), contrasting conditions (i.e., sampling pools characterized by contrasting geochemistry or characteristics; e.g., Meyer-Dombard et al., 2005; Lindsay et al., 2019) or transect across the subsurface to surface transition along temperature or redox gradients (i.e., surface mixing gradients; e.g., Giovannelli et al., 2013; Sánchez-Murillo et al., 2014; Brazelton et al., 2017; Fones et al., 2019). These approaches involve sample collection at the centimeter to tens of meter scale, often using control sites to investigate the role of microbial communities in the setting under investigation. While this approach has deep roots in ecology and microbial ecology, it often limits our ability to comprehend the response of the community to the underlying geological processes, for example concluding that temperature and sulfide gradients drive microbial community assembly (Giovannelli et al., 2013). On the contrary, studies focusing on large-scale systematic investigations of the response of deep subsurface communities to geological gradients allow for conclusions to extend to direct links with geological processes (Crossey et al., 2016; Power et al., 2018; Colman et al., 2019a; Cascone et al., 2021; Fullerton et al., 2021; Rogers et al., 2022).

**Figure 2:**
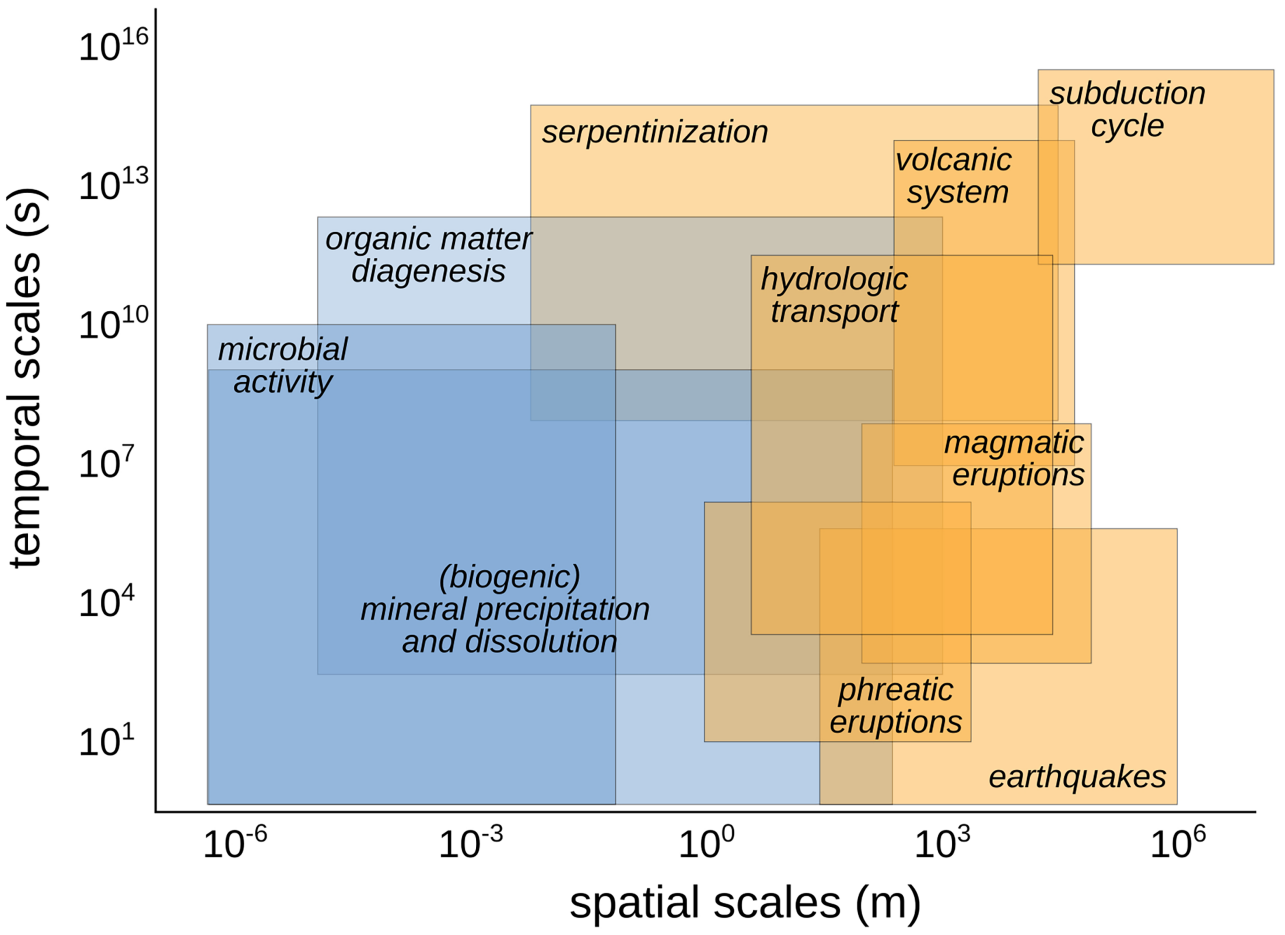
Conceptual diagram showing the relative spatial and temporal scales of biological (in blue) and geological (in orange) processes at convergent margins and their overlap in space–time. Similar conceptual diagram showing the overlap between geological and biological processes in different tectonic settings can help to identify the relevant processes to track and the type of synoptic, co-located samples to collect.

One of the barriers in approaching the study of microbial processes at geologically relevant scales is the lack of understanding of temporal and spatial scales at which these processes overlap (Figure 2). Recent studies have shown that individual microbes in the subsurface undergo total carbon turnover times spanning months to hundreds of years, meaning that an individual can survive for even longer, possibly operating on timescales beyond thousands of years (Hoehler and Jørgensen, 2013; Lever et al., 2013; Braun et al., 2017; Lloyd, 2021). Any interaction these microbes have with the surrounding fluids and solid mineral phases at depth should then be considered to occur over these long timescales over which the microbes operate. Processes like microbial metabolic transformations of volatiles, the diagenesis of organic matter and the microbially-mediated dissolution and precipitation of minerals might act at temporal and spatial scales that overlap with geological processes in the same area (Figure 2). For example, at convergent margins, subsurface microbial metabolism can significantly impact the quantity of carbon recycled in the outer forearc and forearc regions, while responding cohesively to subduction parameters (Barry et al., 2019; Fullerton et al., 2021).

Identifying the theoretical overlap between geological and biological processes in geological settings is a key step in identifying the relevant geo-bio-processes to track and the type of synoptic, co-located samples to collect. Once identified, large-scale geological gradients can be sampled in a similar fashion to small-scale local gradients. Thus, samples can be collected from deeply-sourced seeps at intervals of tens or hundreds of kilometers along ideal transects crossing the geological region under investigation (Figure 1). This effectively moves the scale of investigation from local to meso- and large-spatial scales, tracking subsurface biosphere properties as they covary with geological processes. As a consequence, the resulting inferences have broad planetary implications, both at the ecosystem level and in deep time. Comparing samples across broad spatial scales may also give insights into how the paleomicrobiological environments (Rivera-Perez et al., 2018; Arning and Wilson, 2020) have changed through time.

### Synoptic and colocated samples from diverse scientific disciplines

One of the keys to identifying biological and geological feedbacks is collecting samples that are synoptic (i.e., collected at the same time) and colocated (i.e., collected on the same location or in the immediate proximity), for as wide a range as possible of biological, chemical and geological parameters. Since individual laboratories are usually not capable of conducting high resolution measurements of disparate geochemical and biomolecular data, this can be only accomplished by bringing together a team of scientists in different fields of research, with the added benefit of enabling cross-cutting connections based on discussions between scientists from different fields. For instance, geologists often collect samples to characterize abiotic processes that are analyzed for concentrations and isotopic composition of gases, aqueous molecules, petrological inclusions, and mineralogy, which are interpreted without explicitly addressing potential biological interactions. On the other hand, microbiologists often assess biotic processes, collecting samples to produce cultures and biomolecular datasets for DNA, RNA, proteins, lipids and metabolites, and interpret them against a limited number of geochemical variables that describe the local environment, without reference to the larger geological context and longer time scale processes.

To bring the problem into focus, it is pertinent to analyze a few examples from well-studied locations such as the deep-sea hydrothermal vents of the East Pacific Rise and the hot springs of Yellowstone National Park. Hydrothermal vents located on the 9°50′N segment of the East Pacific Rise are among the best studied vents in the world, with biological and geochemical observations going back four decades (Jannasch and Wirsen, 1979; Jannasch and Mottl, 1985; Sylvan et al., 2012; Vetriani et al., 2014; Gulmann et al., 2015; McNichol et al., 2018). While the pioneering studies at these locations have tremendously contributed to our understanding of how the microbiology of the hot oceanic subsurface might operate, the number of studies that simultaneously discuss the detailed gas and aqueous geochemistry and microbiology of the same sample are scarce, and typically limited to a handful of vent locations. While sampling large spatial coverage of deep-sea hydrothermal vents is much harder to achieve given the large amount of exploration effort required to discover them, studies carried out at the more accessible Yellowstone National Park hydrothermal system show a similar trend with comparisons of the microbial diversity found in a large number of geochemically-diverse springs (Inskeep et al., 2013; Colman et al., 2019a). Even in these studies, data pertaining to the geochemistry of the gases and isotopic composition is often missing, fragmented, non-standardized, or measured on other samples during different field seasons. Altogether, we argue that the absence of co-located, synoptic samples impairs the ability to make connections among the different geobiological processes on larger spatial and temporal scales.

Collecting a large number of variables on co-located synoptic samples provides unique opportunities. Despite this, it also brings to the table a large number of challenges associated with working with a large number of potentially covarying variables (Fan et al., 2014). Classical correlational approaches can be subjected to large number artifacts (e.g., spurious correlations) when dealing with large datasets. In this case abductive approaches need to be used for hypothesis generation and followed by inductive studies confirming (or rejecting) the generated hypothesis. In doing this, a number of big data approaches can be used, such as large-scale multivariate techniques (Ranjard et al., 2013; Danko et al., 2021; Cordone et al., 2022), network analysis (Delgado-Baquerizo et al., 2018; Fullerton et al., 2021) and machine learning approaches (Ghannam et al., 2020; Fullerton et al., 2021). Although the number of studies employing such techniques is still limited, these approaches provide access to underlying trends in the data structure, and, if carefully interpreted in the light of the existing body of knowledge, can provide fruitful insights.

### Conclusion and future perspectives

We presented here a new rationale to approach the study of subsurface ecosystems through the use of deeply-sourced seeps as windows into the subsurface. Our approach calls for the collection of a large number of colocated synoptic samples across large spatial scales, purposefully ignoring more local and likely surface derived variability.

Specifically our approach can be summarized as follows:

1. Use a combination of pre-existing and newly sampled field data to identify deeply-sourced seeps and samples to minimize surface contamination;
2. Sample across large geologic gradients (hundreds of kms), following changes in geological processes of interest;
3. Use a multi-pronged approach to identify the relative contribution of deep vs surface processes for each sampling site (Table 1). No single perfect proxy exists and the strategy will need to be adapted to the unique features of the system under investigation;
4. Collect geological, geochemical and biological data synoptically, with samples taken as close together as possible (i.e., from the same seep and at the same time) in order to minimize confounding factors (small-scale spatial and temporal variability). Although an inductive, hypothesis driven approach is productive, employing an abductive, data-driven approach can also lead to unanticipated discoveries. Make sure to account for statistical problems that might arise from this, like accounting for multiple hypothesis testing, collinearity of variables and large number of false positives;
5. Analysis should focus on features that covary across the dataset, possibly using large-scale correlational approaches, big data analytics and machine learning. Relationships that are identified between biological and geological processes should be backed up with mechanistic connections between variables (possibly through the presence of known ecosystem role and function, physiologies, metabolic pathways, or bulk activity measurements on natural samples).

Earth’s subsurface is vast, with at least 10^29^ living microbial cells (Kallmeyer et al., 2012; Magnabosco et al., 2018). These microbial communities are some of the most diverse, yet least-described microbial communities in the world (Huber et al., 2006; Santelli et al., 2008; Lloyd et al., 2018), and might be relevant to understand not only the coevolution of life with our planet (Chopra and Lineweaver, 2016; Giovannelli et al., 2020), but also in the search for life in the universe (Parnell and McMahon, 2016; Giovannelli et al., 2021). Many of these populations may be growing very slowly (Hoehler and Jørgensen, 2013), meaning that biological processes may overlap in timescale with slower geological processes. Investigating these overlaps requires a large-scale approach to sampling deep subsurface life, where a large suite of biological and geochemical data are collected simultaneously, often by different laboratories. Given the infeasibility of drilling hundreds of boreholes for a single study, here we describe an approach where sampling of surface-expressed fluids from natural springs across a geological gradient is used to investigate biological and geological feedbacks in Earth’s subsurface. This approach has been used in only a handful of studies thus far, but a broader adoption of these methods may accelerate discoveries about life in the subsurface and how it interacts with geological processes even at planetary level.

## Data availability statement

The original contributions presented in the study are included in the article/supplementary material, further inquiries can be directed to the corresponding author.

## Author contributions

All authors listed have made a substantial, direct, and intellectual contribution to the work and approved it for publication.

## Funding

DG has received funding from the European Research Council (ERC) under the 17 European Union’s Horizon 2020 research and innovation programme (grant 18 agreement no. 948972) ERC-STG-2020 project CoEvolve. Additional support came from The National Fund for Scientific and Technological Development of Chile (FONDECYT) Grant 11191138 (The National Research and Development Agency of Chile, ANID Chile), COPAS COASTAL ANID FB210021 to GJ. KL, JM, and PB were supported by the NSF-FRES award 2121637. Partial support came from the Alfred P. Sloan Foundation and the Deep Carbon Observatory (G-2016-7206) to PB, JM, DG, and KL. Additional support came from Simons Foundation 404586 to KL.

## Conflict of interest

The authors declare that the research was conducted in the absence of any commercial or financial relationships that could be construed as a potential conflict of interest.

## Publisher’s note

All claims expressed in this article are solely those of the authors and do not necessarily represent those of their affiliated organizations, or those of the publisher, the editors and the reviewers. Any product that may be evaluated in this article, or claim that may be made by its manufacturer, is not guaranteed or endorsed by the publisher.
